# Supplementary material for: Geniposide ameliorates bleomycin-induced pulmonary fibrosis in mice by inhibiting TGF-β/Smad and p38MAPK signaling pathways
Source: PLoS One. 2024 Sep 6;19(9):e0309833. doi: 10.1371/journal.pone.0309833 (PMC11379225; doi:10.1371/journal.pone.0309833)
Supplement: S1 Raw images — (PDF) [file pone.0309833.s001.pdf]

Supplementary Figures

Geniposide ameliorates bleomycin-induced pulmonary fibrosis in mice by inhibiting TGF-β/Smad and p38MAPK signaling pathways

Jian-Bin Yin [1,2¶], Ying-Xia Wang[3¶], Su-Su Fan[2¶], Wen-Bin Shang[2], Yu-Shan Zhu [2], Xue-Rong Peng [2], Cheng Zou [2*], Xuan Zhang [2*]

Fig 6. Geniposide（Gen） significantly down-regulated the expression of TGF-β1and Smad2/3 in lung tissue of bleomycin-induced PF mice.

Figure 6-A

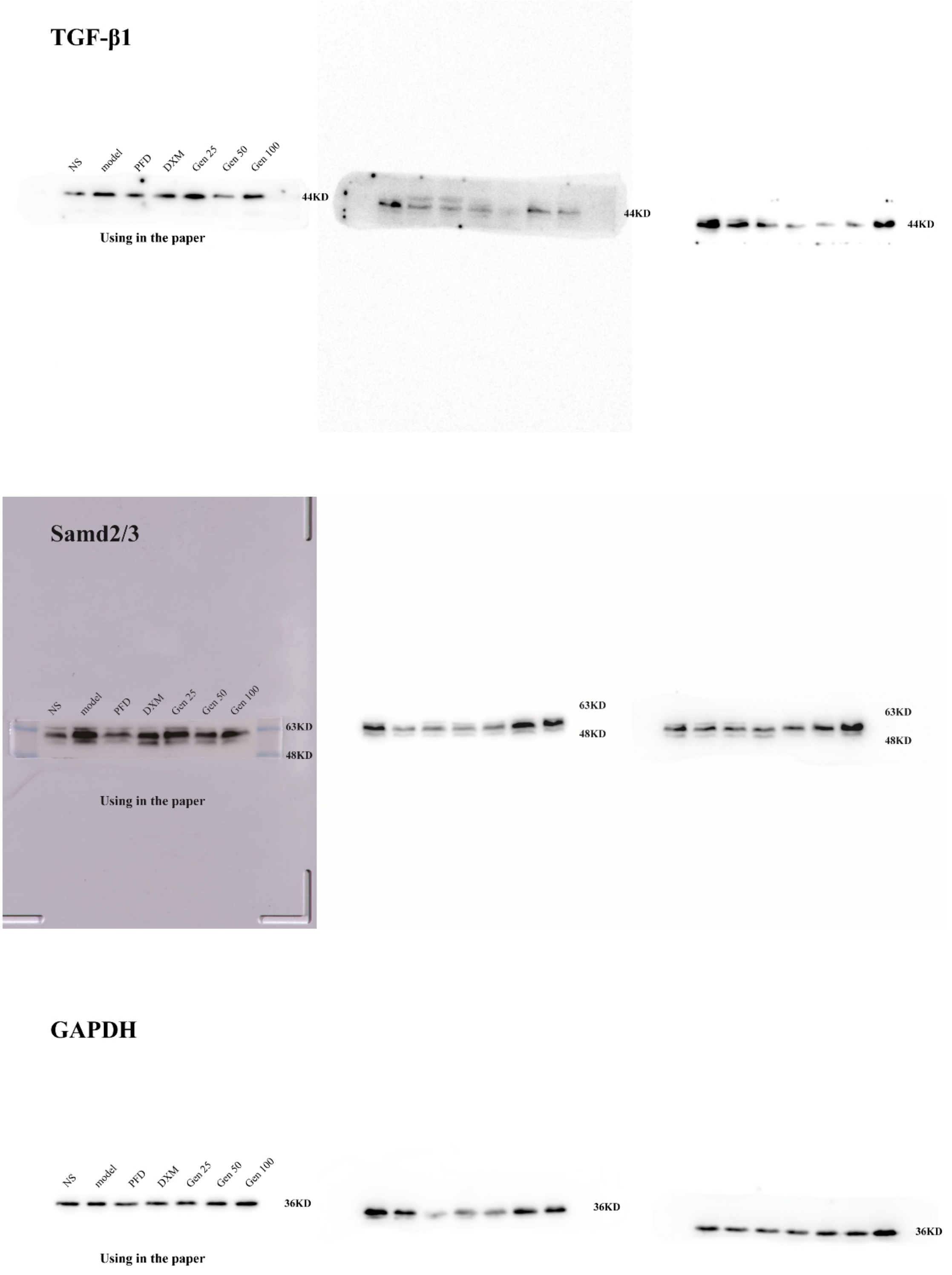

Figure 6-D

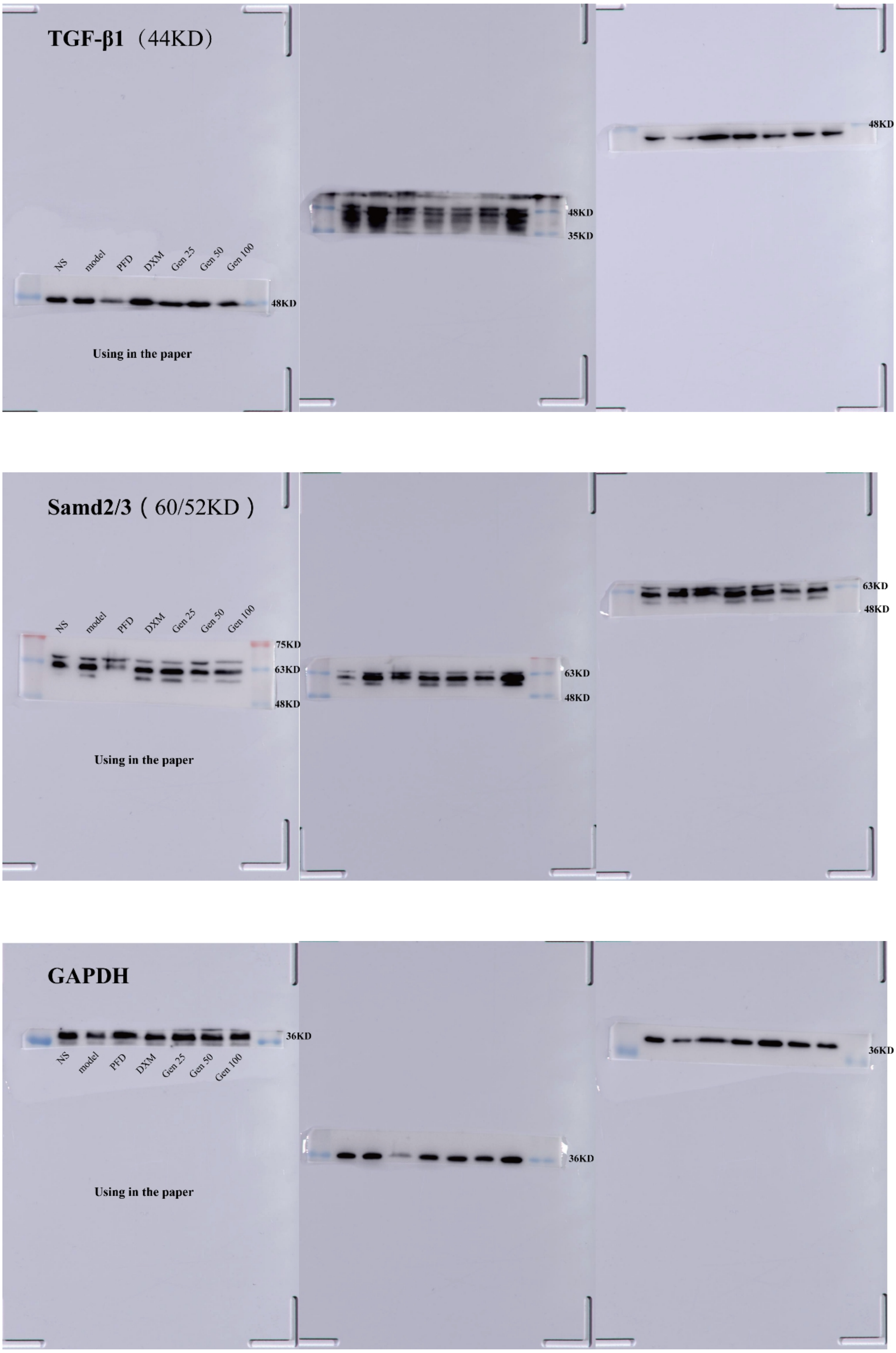
TGF-β1（44KD）
NS
model
PFD
DXM
Gen 25
Gen 50
Gen 100
48KD
Using in the paper
48KD
35KD
48KD
Samd2/3 (60/52KD)
NS
model
PFD
DXM
Gen 25
Gen 50
Gen 100
75KD
63KD
48KD
Using in the paper
63KD
48KD
63KD
48KD
GAPDH
36KD
NS
model
PFD
DXM
Gen 25
Gen 50
Gen 100
Using in the paper
36KD
36KD

Fig 7. Geniposide significantly down-regulated the expression of CTGF and p38 in lung tissue of bleomycin-induced PF mice.

Figure 7-A

CTGF

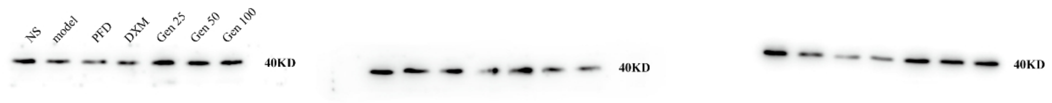

Using in the paper

P38

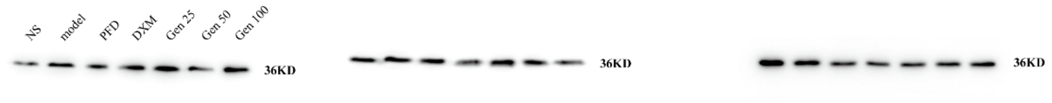

Using in the paper

GAPDH

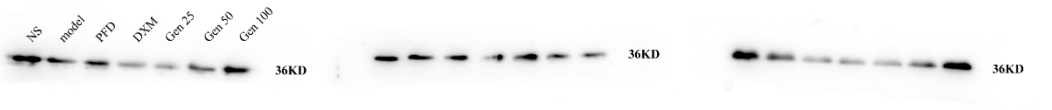

Using in the paper

Figure 7-D

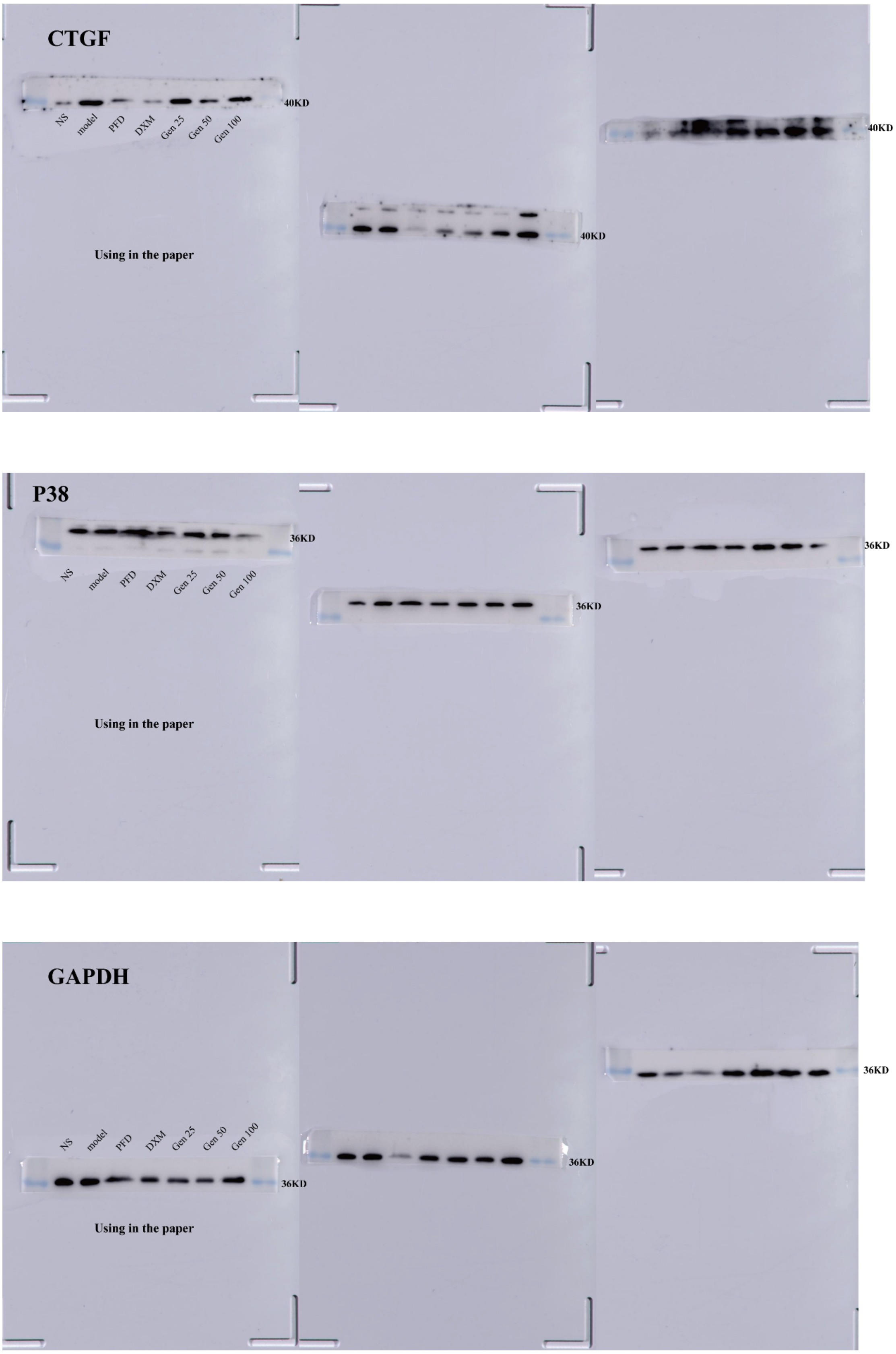
CTGF
40KD
NS
model
PFD
DXM
Gen 25
Gen 50
Gen 100
Using in the paper
40KD
40KD
P38
36KD
NS
model
PFD
DXM
Gen 25
Gen 50
Gen 100
Using in the paper
36KD
36KD
GAPDH
NS
model
PFD
DXM
Gen 25
Gen 50
Gen 100
36KD
Using in the paper
36KD
36KD
