# Supplementary figures and images for: Geniposide ameliorates bleomycin-induced pulmonary fibrosis in mice by inhibiting TGF-β/Smad and p38MAPK signaling pathways
Source: PLoS One. 2024 Sep 6;19(9):e0309833. doi: 10.1371/journal.pone.0309833 (PMC11379225; doi:10.1371/journal.pone.0309833)

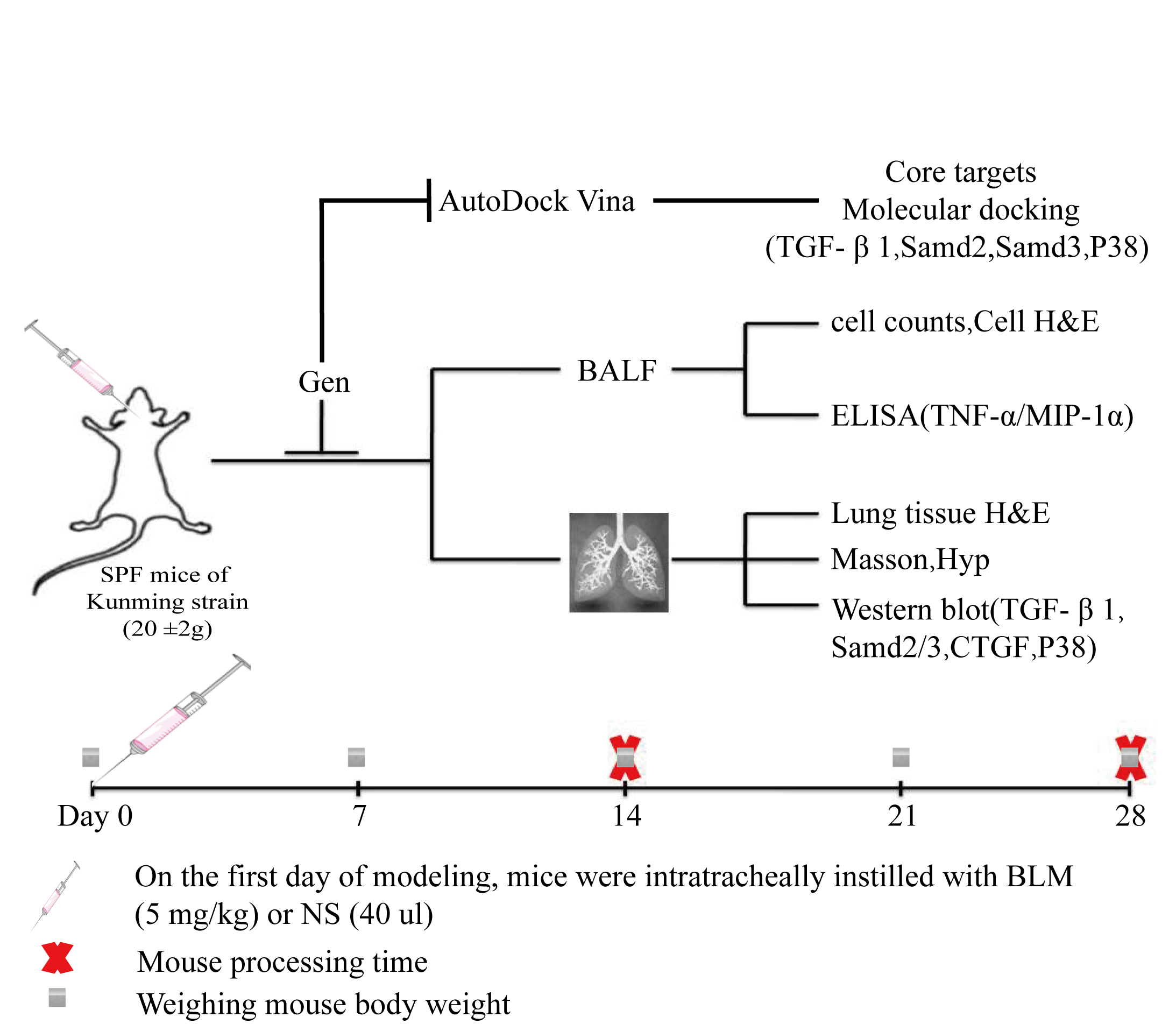

Supplement: S1 Fig — (TIF) [file pone.0309833.s002.tif]

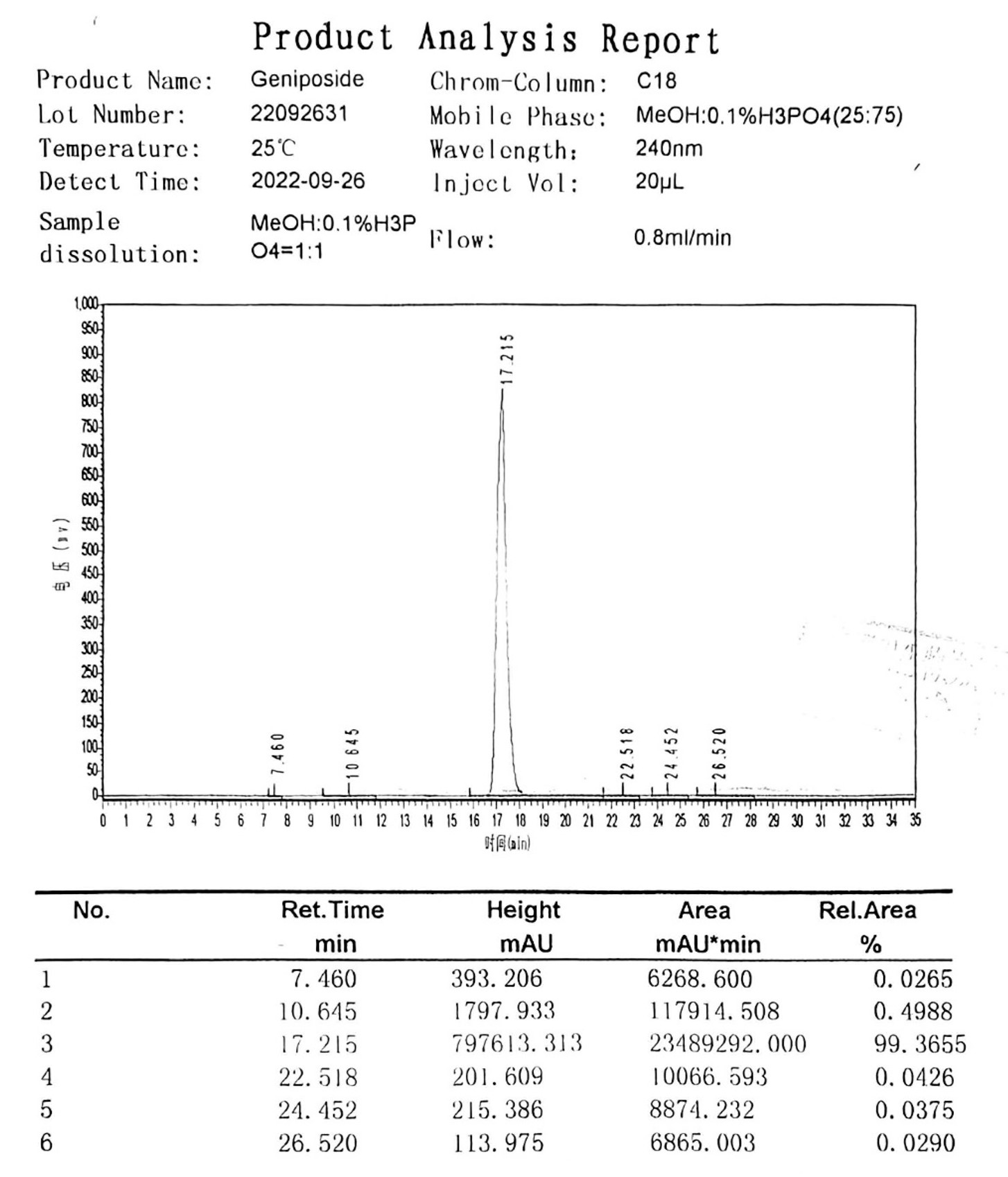

Supplement: S2 Fig — (TIF) [file pone.0309833.s003.tif]
