# Supplementary material for: Geniposide ameliorates bleomycin-induced pulmonary fibrosis in mice by inhibiting TGF-β/Smad and p38MAPK signaling pathways
Source: PLoS One. 2024 Sep 6;19(9):e0309833. doi: 10.1371/journal.pone.0309833 (PMC11379225; doi:10.1371/journal.pone.0309833)
Supplement: S1 File — (PDF) [file pone.0309833.s005.pdf]

Pulmonary histopathology- Alveolar inflammation and pulmonary fibrosis score

The stained sections were observed and photographed under the Niko DS-Ri2 automatic microscope for evaluation of alveolar inflammation and pulmonary fibrosis. Ordinal data is expressed in the form of evaluation scores: (-) is 1 point, (+) is 2 points, (++) is 3 points, and (+++) is 4 points.The specific standards are as follows:

Alveolar inflammation score:

Perform H&E staining on lung tissue slices of each experimental group, followed by observing and scoring alveolar inflammation to quantify the degree of alveolar inflammation. As follows:

Level 0: No alveolitis (-), no thickening of alveolar walls, normal alveolar structure, and no visible large alveoli.

Level 1: Mild (+), thickening of the alveolar septum by a mononuclearcell infiltrate, with involvement limited to focal,pleural-based lesions occupying less than 20 % of thelung and with good preservation of the alveolararchitecture.

Level 2: Moderate (++), a more widespread alveolitis involving 20 to 50 % of the lung, althoughstill predominantly pleural based.

Level 3: Severe (+++), adiffuse alveolitis involving more than 50 % of thelung, with occasional consolidation of air spaces bythe intra-alveolar mononuclear cells and some hemorrhagic areas within the interstitium and /or alveolus.

pulmonary fibrosis score:

Masson staining was performed on lung tissue slices of each experimental group, followed by observation and evaluation of pulmonary interstitial fibrosis score to quantify the degree of pulmonary fibrosis. As follows:

Level 0: Normal lung tissue (-), no evidence of fibrosis.

Level 1: Mild fibrosis (+), focal regions offibrosis involving less than 20 % of the lung. Fibrosisinvolved the pleura and the interstitium of the subpleural parenchyma with some distortion of alveolar architecture.

Level 2: Moderate fibrosis (++), presenting with extensive fibrosis, moderate accumulation of collagen fibers, and lesion areas accounting for 20% to 50% of the entire lung. At this time, the alveolar structure is disordered, but fibrosis still only occurs

locally.

Level 3: Severe fibrosis (+++), widespread fibrosis, involving more than 50 % of the lung. Confluent lesionswith extensive derangement of parenchymal architecture, including cystic air spaces lined by cuboidalepithelium..

Reference:

Szapiel SV, Elson NA, Fulmer JD, Hunninghake GW, Crystal RG. Bleomycin-induced interstitial pulmonary disease in the nude, athymic mouse. Am Rev Respir Dis. 1979 Oct;120(4):893-9. doi: 10.1164/arrd.1979.120.4.893. PMID: 92208.

Example:

1、 Alveolitis:

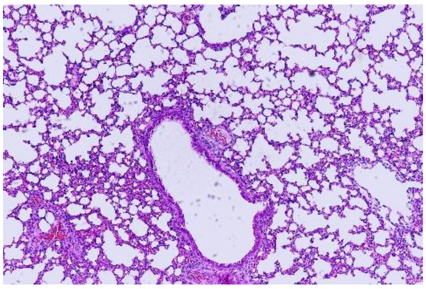

Level 0: No alveolitis (-)

Score：1

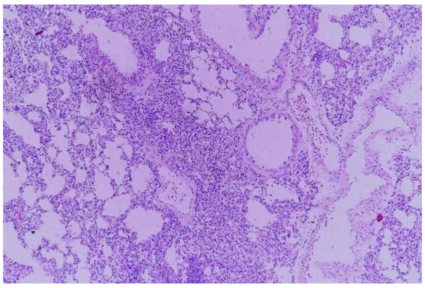

Level 3: Severe (+++)

Score：4

2、 Fibrosis:

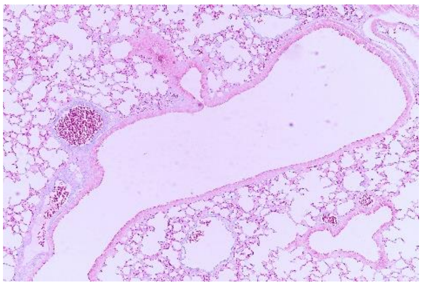

Level 0: Normal lung tissue (-)

Score：1

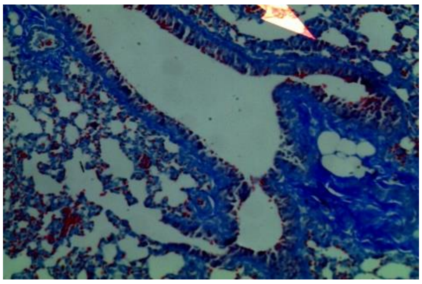

Level 3: Severe fibrosis (+++)

Score：4

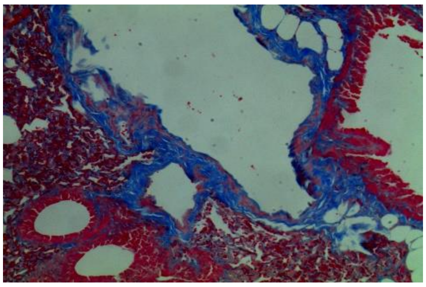

Level 2: Moderate fibrosis (++)

Score：3
