# Supplementary material for: Geniposide ameliorates bleomycin-induced pulmonary fibrosis in mice by inhibiting TGF-β/Smad and p38MAPK signaling pathways
Source: PLoS One. 2024 Sep 6;19(9):e0309833. doi: 10.1371/journal.pone.0309833 (PMC11379225; doi:10.1371/journal.pone.0309833)
Supplement: Attachment — Submitted filename: Response to Reviewers.docx [file pone.0309833.s006.docx]

**Response to reviewers**

**Review comments**

**Reviewer #1:**

1) The specific dosages and treatment duration of Geniposide are not provided in abstract.

**Response(R):The modifications have been completed. Please refer to the red font section in the “Abstract”.**

2) Insufficient justification for Geniposide selection: The introduction mentions that Geniposide has various pharmacological activities, including anti-inflammatory effects, but it does not provide a strong justification for selecting Geniposide as a potential therapeutic agent for PF. A more detailed explanation of the rationale behind this choice would strengthen the introduction.

**R:The modifications have been completed. Please refer to the red font section in the “Introduction:**“Our previous research found that Geniopicoside, which also has **anti-PF** effects[25], has a similar structure to Gen and belongs to the class of iridoid glycosides. However, Gen has a more stable structure and is easier to obtain in nature than Geniopicoside. Based on these, we hypothesized that Geniposide may have a potential therapeutic effect on PF.”

3) The description of the histopathology scoring method is incomplete. It refers to the scoring method of Szapield and Fulmer et al., but the specific criteria used for grading alveolitis and fibrosis are not provided. Including the criteria and providing examples would enhance the reproducibility of the results.

**R:** **The histopathological scoring method has been added to the "Supporting information" section. Please refer to the "S4 Histopathology scoring method"**

4) In the 2.10 Molecular docking section, add more details. For example, 90&times;90&times; 90&Aring; (x, y, and z) grid box protease binding pocket with XXX nm spacing for each dimension and a grid center at dimensions of XXX, YYY and ZZZ.

**R: The details of docking parameters have been added, Please refer to 2.10 Molecular docking section.**

5) In result section, add degree of freedom for data as well as exact p value. For example: [F(4,36)=100, p=0.003]

**R:** **The modifications have been completed.**

6) First paragraph of discussion just mention the most important findings.

**R:The modifications have been completed. Please refer to the first paragraph of the "Discussion" section**

7) Additional limitations of the study design could be acknowledged, such as the use of only male mice. Discussion of these limitations would add balance.

**R: The modifications have been completed. Please refer to the second paragraph of the "Discussion" section**

8) Compare more about 14th day and 28th days.

**R:** **It cannot be directly compared because 14 and 28 days are animals euthanized in different time batches, and they are in different stages of pulmonary fibrosis disease. The pulmonary fibrosis model mice were euthanized from the first day of tracheal instillation of bleomycin until the 28th day, which was a continuous and progressive process. In the experimental design of this study, lung tissue was selected to be taken on the 14th and 28th days respectively, because on the 14th day, inflammation was mainly present in the lung tissue and it was a stage of transition toward fibrosis; On the 28th day, significant fibrosis had formed in the lung tissue, and the inflammatory response had gradually subsided, manifested as chronic inflammation.**

Reference:+

[1]Liu T, De Los Santos FG, Phan SH. The Bleomycin Model of Pulmonary Fibrosis. Methods Mol Biol. 2017;1627:27-42. doi: 10.1007/978-1-4939-7113-8_2. PMID: 28836192.

9) The writing could be tightened in places to increase clarity and readability. For example, some sentences are quite long. Breaking them up may help.

**R: Some long sentences have been break into short sentences.**

**Reviewer #2:**

**Major issues:**

1. They claimed there are no reports of whether geniposide has a protective effect against pulmonary fibrosis (page 5, line 97), which is not true. Yi Wei et al reported similar effect and published their results in Journal of Functional Foods, Volume 104, May 2023, 105503. They found that geniposide improves bleomycin-induced pulmonary fibrosis by inhibiting NLRP3 inflammasome activation and modulating metabolism. Zhang will need to discuss and compare the difference/novelty between their study with this study.

**R: Thanks for kindly reminding us of this reference, we didn’t find this reference before. We have deleted the claim of no reports of whether geniposide has a protective effect against pulmonary fibrosis. And we discussed and compared the difference/novelty between our study and this reference in the Discussion Section.**

2. ①They conclude from H&amp;E staining that geniposide (Gen) significantly reduces the number of inflammatory cells, including macrophage (M), granulocyte (G) and lymphocyte. The quantification for this data is missing. ②They should use arrows to point out examples of macrophage (M), granulocyte (G) and lymphocyte in their images. ③Immunostaining of marker genes for these immune cells will strengthen their findings. ④Also, resident macrophages change their morphology upon activation. Morphological characterization of the morphology macrophages will help as well.

**R**①**:In the original paper version, Fig 4 quantified the number of inflammatory cells in BALF, where Fig 4-A and B represent the total number of inflammatory cells in BALF, and Fig 4-C and D represent the classification and counting results of three types of inflammatory cells (M/G/L).**

**In the latest revised manuscript, we have merged Fig 3 and Fig 4 together. Please refer to "Fig 3" in the revised manuscript.**

**R②: The modification has been completed, please refer to "Fig3"**

**R**③**:Sorry, for the convenience of observation and preservation under the microscope after staining the cell smear, we have undergone resin sealing treatment and cannot conduct further experiments; The cycle of preparing animal models again is relatively long.**

**R**④**:The modifications have been completed. We have described the morphology of macrophages after activation, please refer to the fifth paragraph of the "Discussion" section**

3. They showed that geniposide reduces inflammation and bleomycin-induced idiopathic pulmonary fibrosis. Does geniposide directly act on immune cells to inhibit their reactivity, which then contributes to reduction of fibrosis? In vitro culture of macrophages treated with geniposide can support this hypothesis.

**R: We have supplemented the in vitro study results. We observed the effect of geniposide on LPS-induced Raw264.7 macrophages and found that geniposide significantly changed the morphology of LPS induced Raw264.7 macrophages and inhibited the levels of IL-1β and TNF-α in cell supernatants，suggesting that geniposide could directly inhibit the activation of macrophage induced by LPS. please refer to “2.11 In vitro experiment” and “3.9 Effect of Geniposide on the activation of Raw 264.7 cells induced by LPS”**

**Minor issues:**

1. HPLC curves to show the purity of geniposide should be provided.

**R: The HPLC curves were provided in the supporting information(S5).**

2. Quantification results should be in the same figure with their representative. This applies to figure 1 to 3.

**R:We have merged Fig3 and Fig4 from the old version together, please refer to "Fig3" in the revised manuscript.**

3. Representative image are usually put in front of quantification results. This applies to fig.6 to 7.

**R:** **The modification has been completed, please refer to "Fig6, 7"**

4. Molecular docking predicts geniposide binds to TGF-beta;1, Smad2, Smad3, and p38. Are their any proteins which geniposide does not bind? Showing a few examples of proteins which geniposdie does not bind will highlight the specificity of this drug.

**R:** **In molecular docking, the binding energy score is used to evaluate the binding specificity of drug to the target proteins. The binding energy score criterion has been added in the 2.10 Molecular docking section. And in the 3.8 Molecular docking section, we added “The binding energy score was used to evaluate the binding specificity, and a score less than -5 was usually a criterion for specific binding. The binding energy scores of TGF-β1, Smad2, Smad3, and p38 were -8.6, -6.2, -6.8 and -6.9, respectively. ”**

5. Error bars should be provided in staining images. (Figure 1 and 2)

**R:** **We guess the reviewer meant the scale bars， and have added scale bars in** in staining images **（Fig2 and Fig3）**

6. Are there any bad effects of geniposide treatment, such as cell death, tissue damage, or organ failure?

**R:** **An article reported an acute toxicity test of geniposide [1,2], which showed that liver toxicity was only induced when the dose of geniposide reached 574mg/kg in rats. In another study [3], the authors induced a Kunming strain mouse liver injury model with different doses of geniposide; The results showed that when the dose reached 420mg/kg, there was a difference in serum ALT and AST levels between the mice and the normal group. From the above evidence, it can be seen that the dose range used in this study is safe, and no adverse reactions were observed during our research process.**

[1]Ding, Y.; Zhang, T.; Tao, J.S.; Zhang, L.Y.; Shi, J.R.; Ji, G. Potential hepatotoxicity of geniposide, the major iridoid glycoside in dried ripe fruits of Gardenia jasminoides (Zhi-zi). Nat. Prod. Res. 2013, 27, 929–933.

[2]Shan, M.; Yu, S.; Yan, H.; Guo, S.; Xiao, W.; Wang, Z.; Zhang, L.; Ding, A.; Wu, Q.; Li, S.F.Y. A Review on the Phytochemistry, Pharmacology, Pharmacokinetics and Toxicology of Geniposide, a Natural Product. Molecules 2017, 22, 1689. <https://doi.org/10.3390/molecules22101689>

[3] Liu, Q.; Lu, B.P.; Jia, R. Preliminary study on the establishment of mouse model of liver injury by perfusing stomach with geniposide. China J. Chin. Med. 2013, 28, 994–996.
