# Supplementary material for: Geniposide ameliorates bleomycin-induced pulmonary fibrosis in mice by inhibiting TGF-β/Smad and p38MAPK signaling pathways
Source: PLoS One. 2024 Sep 6;19(9):e0309833. doi: 10.1371/journal.pone.0309833 (PMC11379225; doi:10.1371/journal.pone.0309833)
Supplement: Attachment — Submitted filename: Response to Reviewers.docx [file pone.0309833.s007.docx]

**Response to Reviewers**

**Reviewer # 2 question：**

1. Figure 2: **①**Scale bars are not very visible and appear too thin. Consider using thicker lines and red instead of black for better contrast. **②**Additionally, please clarify what the red feature is in the H&E staining of the PFD group in the right panel as this feature seems unique to the PFD group.

**R：**

**①According to the suggestion, we use a red bold scale for identification**

**②We speculate that you are referring to the staining images of the PFD group in the Pathological H&E staining section of Fig 2 on day 28. The red features in this image represent pulmonary vessels; After H&E staining, the vascular contents (platelets, etc.) were stained red.It should be emphasized that this red feature is not unique to the PFD group, but when selecting the field of view under the microscope, other experimental groups happened to avoid the pulmonary vessels.**

2. Figure 3: **①**It remains unclear how different types of immune cells are recognized in the H&E staining. Please include the criteria used for this seperation in the Methods section. **②**Additionally, the total number of cells in the model group of BALF doubles at 28 days compared to 14 days, but the number of immune cells (M, L, G) does not reflect this change. Please discuss which cell types contribute to this significant increase in cell numbers.

**R：**

**①We mentioned in the footnote at Fig 3 of the manuscript: 'Based on the morphological characteristics of macrophage (M), granulocyte (G) and lymphocyte (L) nuclei, the number of three types of inflammatory cells (M/G/L) in each photograph was counted and the average was calculated (Set to b_M/G/L_)'. We have provided a detailed introduction to the criteria for identifying different immune cells in the "Methods" section of the manuscript.**

**②Thank you for reading our article thoroughly and asking such insightful questions. Our explanation is that in Fig 3, B-C represents the total number of cells actually counted in BALF; In the quantitative analysis of three types of cells (M/G/L) (D-E), in order to reduce bias, we use the proportion of three types of cells (M/G/L) to the total number of cells to represent the results. Since this value is calculated by the ratio of the number of immune cells (a) [M/G/L] to the total number of cells (b) during the same period (14/28 days) (i.e. a/b), the differences in the number of immune cells at different periods cannot be reflected in the histogram D \ E.**

3. Figure 6: Please explain why there is a significant increase in TGF-beta levels in the Gen25 group compared to the model group, while Gen50 shows a reduction in TGF-beta levels.

**R：In Fig 6, B represents the expression of TGF beta in lung tissue on day 14. From the graph, it can be visually observed that the Gen 25 group appears to be significantly upregulated compared to the model group, but after statistical calculations, we found that the difference between the two groups was not significant (P>0.05); Therefore, it cannot be considered that the Gen 25 group is significantly upregulated. As for the downregulation of Gen 50 group expression, we believe it is due to the action of the drug.**

**The location of this revision in the manuscript is:**

1. The "Methods" section of the manuscript has added descriptions of the cell morphology of three types of inflammatory cells

2. The 'Project administration' has been changed in the Author contributions section

3. Changed the citation format of references 6 and 8; Replaced reference 31 with new literature.
